# Radiation Induces Valvular Interstitial Cell Calcific Response in an *in vitro* Model of Calcific Aortic Valve Disease

**DOI:** 10.3389/fcvm.2021.687885

**Published:** 2021-08-30

**Authors:** Manon Meerman, Rob Driessen, Nicole C. A. van Engeland, Irith Bergsma, Jacco L. G. Steenhuijsen, David Kozono, Elena Aikawa, Jesper Hjortnaes, Carlijn V. C. Bouten

**Affiliations:** ^1^Department of Cardiothoracic Surgery, Heart and Lung Division, Leiden University Medical Center, Leiden, Netherlands; ^2^Department of Biomedical Engineering, Soft Tissue Engineering and Mechanobiology (STEM), Eindhoven University of Technology, Eindhoven, Netherlands; ^3^Institute for Complex Molecular Systems (ICMS), Eindhoven University of Technology, Eindhoven, Netherlands; ^4^Åbo Akademi University, Faculty of Science and Engineering, Molecular Biosciences, Turku, Finland; ^5^Department of Radiotherapy, Catherina Ziekenhuis Eindhoven, Eindhoven, Netherlands; ^6^Department of Radiation Oncology, Dana-Farber Cancer Institute and Harvard Medical School, Boston, MA, United States; ^7^Center for Interdisciplinary Cardiovascular Sciences, Cardiovascular Medicine, Brigham and Women's Hospital and Harvard Medical School, Boston, MA, United States

**Keywords:** aortic valve disease, radiotherapy, extracellular matrix, *in vitro* modeling, valvular interstitial cells

## Abstract

**Background:** Mediastinal ionizing radiotherapy is associated with an increased risk of valvular disease, which demonstrates pathological hallmarks similar to calcific aortic valve disease (CAVD). Despite advances in radiotherapy techniques, the prevalence of comorbidities such as radiation-associated valvular disease is still increasing due to improved survival of patients receiving radiotherapy. However, the mechanisms of radiation-associated valvular disease are largely unknown. CAVD is considered to be an actively regulated disease process, mainly controlled by valvular interstitial cells (VICs). We hypothesize that radiation exposure catalyzes the calcific response of VICs and, therefore, contributes to the development of radiation-associated valvular disease.

**Methods and Results:** To delineate the relationship between radiation and VIC behavior (morphology, calcification, and matrix turnover), two different *in vitro* models were established: (1) VICs were cultured two-dimensional (2D) on coverslips in control medium (CM) or osteogenic medium (OM) and irradiated with 0, 2, 4, 8, or 16 Gray (Gy); and (2) three-dimensional (3D) hydrogel system was designed, loaded with VICs and exposed to 0, 4, or 16 Gy of radiation. In both models, a dose-dependent decrease in cell viability and proliferation was observed in CM and OM. Radiation exposure caused myofibroblast-like morphological changes and differentiation of VICs, as characterized by decreased αSMA expression. Calcification, as defined by increased alkaline phosphatase activity, was mostly present in the 2D irradiated VICs exposed to 4 Gy, while after exposure to higher doses VICs acquired a unique giant fibroblast-like cell morphology. Finally, matrix turnover was significantly affected by radiation exposure in the 3D irradiated VICs, as shown by decreased collagen staining and increased MMP-2 and MMP-9 activity.

**Conclusions:** The presented work demonstrates that radiation exposure enhances the calcific response in VICs, a hallmark of CAVD. In addition, high radiation exposure induces differentiation of VICs into a terminally differentiated giant-cell fibroblast. Further studies are essential to elucidate the underlying mechanisms of these radiation-induced valvular changes.

## Introduction

Despite ongoing technical advances, radiotherapy for treating malignancies in the thoracic region, including lung cancer, thymoma, and lymphoma, is still associated with an increased risk of cardiovascular disease (1, 1–4). As a consequence of exposure of the heart and great vessels to radiation, patients receiving radiotherapy have an increased risk of developing pericarditis, coronary artery disease, cardiomyopathy, and valvular disease years after initial exposure (2, 3, 5–8). Moreover, this risk is even higher in patients receiving radiotherapy before the age of 21 (5, 9). Aortic valve disease represents the majority of radiation-induced valvular diseases (4, 10, 11). Within 20 years after mantle radiation for lymphoma, the incidence of aortic valve stenosis is as high as 16% (12). The only effective treatment for aortic valve stenosis is surgical replacement of the diseased valve (13), however, surgical risks are significantly increased in patients that received chest radiotherapy (14).

Radiation-associated aortic valve disease demonstrates pathological hallmarks similar to calcific aortic valve disease (CAVD) (11, 15, 16). Generally, CAVD is characterized by initial fibrotic thickening of the aortic valve leaflets, which progresses into mineralization of the valve and eventually causes aortic valve leaflet dysfunction due to aortic valve stenosis (17). Traditionally, CAVD was believed to be a passive disease, culminating from years of wear and tear. However, developing insights into the disease have changed these views, and CAVD is now considered to be an active disease process (17). One of the key players in CAVD are resident valvular interstitial cells (VICs) (18). In the normal, healthy aortic valve, VICs represent quiescent fibroblast-like cells, which can differentiate into myofibroblast-like cells upon exposure to environmental stimuli (18, 19). These activated VICs, characterized by alpha smooth muscle actin (αSMA) expression, can remodel the extracellular matrix (ECM) by expressing matrix metalloproteinases (MMPs) or depositing ECM proteins such as collagen (19). The interplay between the quiescent and activated state of VICs is essential in maintaining valvular tissue homeostasis, and provides for the ability of heart valves to adapt to changes in functional demand (20, 21).

The fibrocalcific response observed in early CAVD is thought to be a consequence of persistent activation of VICs (18). In addition, histological studies of end-stage stenotic aortic valves have demonstrated the presence of osteoblast-like cells (21, 22). Numerous reports demonstrated that activated VICs can also differentiate into osteoblast-like cells and actively deposit calcium in the valve interstitium (21). This osteoblast-like differentiation is characterized by the loss of the αSMA expression and the elevation of bone markers, including the transcription factors Runx2 and Osterix, and increased alkaline phosphatase (ALP) activity (23). The temporal relationship of these VIC phenotypes and how they relate to CAVD onset or even progression in humans is not completely understood (20, 21, 24).

Radiation-associated valvular disease cannot merely be equated to CAVD. Specific cellular and ECM responses to ionizing radiation used in radiotherapy have been well documented. Exposure to radiation has shown to activate stromal fibroblasts (25). In addition, increased activation and production of transforming growth factor beta (TGFβ) has been reported as a result of radiation exposure to vessels and myocardium, which in turn leads to increased collagen deposition (26, 27). Explanted heart valves have shown the presence of terminally differentiated atypical giant-cell like fibroblasts, which also have been observed in the skin after radiation therapy (28, 29).

Despite ongoing advances in radiotherapy procedures, the incidence of radiation-induced CAVD is still expected to rise in the future. New radiotherapy techniques are efficient in sparing the heart from radiation exposure while delivering adequate radiation doses to the target area, but improved treatments also increase the number of long-time survivors, thus increasing the number-at-risk (3, 4, 30, 31). To our knowledge, only one other study examined the effects of radiation on the aortic valve in cell culture, but no studies to date have been conducted using three-dimensional (3D) cell culture models of CAVD (32). Such models allow for systematic manipulation of experimental conditions, including radiation dose, and detailed monitoring and analysis of the radiation effects. The aim of this work is to investigate the relationship between radiation and the development of CAVD, using two-dimensional (2D) and three-dimensional (3D) *in vitro* models of the disease. We hypothesize that radiation catalyzes the calcific response of VICs. The work presented here is an initial step in the ongoing research focused on unraveling the effects of radiation exposure on the development of CAVD.

## Materials and Methods

To investigate the effects of radiation on aortic valvular cells and tissue mimics, two *in vitro* models were used. First, the effects of radiation were evaluated on 2D samples containing VICs. VICs, isolated from porcine aortic valves, were cultured on coverslips in either control medium (CM) or osteogenesis permissive medium (OM) and exposed to increasing doses of radiation. Afterwards, cells were kept in culture and analyzed for cell viability and proliferation by measuring adenosine triphosphate (ATP) and DNA content. VIC morphology and differentiation were assessed with RT-PCR and immunohistochemical staining. Osteogenesis in the irradiated VICs was determined by measuring ALP activity. Next, to better mimic the microenvironment of the cells, a methacrylated hyaluronic acid (HAMA) – methacrylatedgelatin (GelMA) 3D hydrogel system was designed and seeded with porcine VICs. 3D hydrogels, cultured in either CM or OM, were then exposed to 0, 4, or 16 Gy of radiation. Experimental setup of the 3D experiments, including the different radiation doses used, were based on data from the 2D experiments. After irradiation of the cell-laden 3D hydrogel constructs, a compaction assay was performed to determine the effect of radiation on hydrogel structure. DNA content was measured to assess cell viability. The hydrogels were cut into sections and stained for DNA damage and various markers to evaluate VIC differentiation. Matrix turnover was analyzed by measuring MMP-2 and MMP-9 activity, measuring gene expression of collagen-encoding genes and immunofluorescent stainings of collagen expression.

### Valvular Interstitial Cell Isolation and Culture

Porcine aortic VICs were isolated from porcine aortic valve leaflets by sequential collagenase/elastase digestion as described previously (33). The cells were cultured in normal growth medium containing Dulbecco's Modified Eagle Medium (DMEM) with high glucose and pyruvate (Gibco, Life Technologies, Grand Island, NY, USA), supplemented with 10% fetal bovine serum (FBS) and 1% penicillin/streptomycin (P/S) (both Gibco) at 37°C and 5% CO_2_. This medium condition was also used as CM. The cells were cultured until 70–80% confluency was reached, and then passaged. For all experiments, cells between passage three and six were used. For immunofluorescence, cells were seeded on gelatin-coated coverslips. Cells from three different donors were used for the 2D experiments and another three donors were used for the 3D hydrogel experiments.

### Hydrogel Fabrication

For the 3D experiments, hybrid hydrogels were fabricated from HAMA and GelMA as reported previously (33), using photo-crosslinking. Briefly, VICs were resuspended in the prepolymer solution, consisting of 1 wt% HAMA and 5 wt% GelMA, in a concentration of 10 million cells/mL. Then, 50 μl of the cell-laden polymer solution was added between two spacers with a height of 450 μm. The cell-laden polymer solutions were then subjected to UV light (wavelength 360 nm) of 2.5 mW/cm^2^ for 30 seconds (Omnicure S2000, EXFO Photonic Solutions Inc, Ontario, Canada). The resulting VIC-laden hydrogels were then transferred to well plates for culturing in either CM or OM. OM consisted of CM supplemented with 10 mM β-glycerophosphate, 10 ng/mL ascorbic acid, and 10 nM dexamethasone (Sigma-Aldrich, St. Louis, MO, USA). The effects of radiation on hydrogel composition were evaluated by performing a compaction assay.

### Irradiation

For the 2D irradiation experiments, VICs were plated at a seeding density of 10.000/cm^2^ 24 h before irradiation. Prior to irradiating the cells, medium was changed to CM or OM. The cells were irradiated with 0, 2, 4, 8, and 16 Gy (0.90 Gy/min) with a Gammacell 40 Exactor Cs-137 irradiator (Best Therotronics, Ottawa, Ontario, Canada). Using the linear-quadratic model (34) with an α/β ratio of 3 Gy for normal tissue late effects, a single 4 Gy dose would be estimated to have the biological effect of a total of 8.5 Gy delivered over 30 fractions, in keeping with modern radiotherapy techniques that seek to lower cardiac dose when treating adjacent organs such as the lung. A single 16 Gy dose would be estimated to have the biological effect of a total of 60 Gy delivered over 30 fractions, which would result from full exposure of the valve to therapeutic doses. After irradiation, cells were kept in culture for 1, 2, 7, or 14 days, depending on the type of analysis.

The 3D hydrogels were fabricated three days before irradiation. Prior to irradiation of the hydrogels, medium was changed to CM with HEPES buffer. The hydrogels were irradiated with 0, 4, and 16 Gy with an Elekta linear accelerator of the hospital. The radiation doses were given from two directions [anterior-posterior-posterior-anterior (AP-PA)] with an instantaneous dose-rate of around 6 Gy/min. The mean dose-rate during irradiation was 4 Gy/min for the 4 Gy group (total treatment time: 1 min) and 5.3 Gy for the 16 Gy group (total treatment time: 3 min). After irradiation, medium was changed to CM or OM and hydrogels were kept in culture for 1, 3, 8, 15, and 21 days, depending on the type of analysis. The control groups of both experiments (0 Gy) were transported to the location of irradiation together with the treated groups.

### Cell Viability and Proliferation

To assess cell viability after irradiation, the ATP content of the cells in the 2D models was measured at day 2 using a luminescent cell viability assay (Progema, Madison, WI). The DNA content was measured in both the 2D and 3D models using the Quant-iT PicoGreen Kit (Life Technologies).

### Histological and Immunofluorescent Stainings

Samples from different time points were used for histological and immunofluorescence analysis. To assess immediate radiation-induced DNA damage in the 3D hydrogels, they were sacrificed 30 to 60 min after irradiation. The hydrogels were fixed in 3.7% formaldehyde solution (Sigma-Aldrich) and kept in 30% sucrose solution overnight at 4°C. The VIC-laden hydrogels were then frozen in optimal cutting temperature (OCT) compound and 10 μm cross-sections were obtained using a cryostat. Sections were treated with peroxide for antigen retrieval, permeabilized with 0.5% Triton X-100 (Sigma-Aldrich) and blocked with 3% bovine serum albumin (BSA) for 30 min. In order to detect radiation-induced DNA-damage, the sections were stained for γH2AX (Ab22551, Abcam), a marker for double-strand DNA breaks, by incubating the sections with anti-γH2AX antibodies for 1 h at room temperature. Sections were counterstained with 4', 6-diamidino-2-phenylindole (DAPI) (Life Technologies) to visualize cell nuclei. Sections were washed, mounted in Mowiol (Sigma-Aldrich) and analyzed using fluorescence microscopy (Axiovert 200M; Carl Zeiss).

At 14 days post-irradiation, VICs in 2D culture were fixed with ice-cold methanol for 30 min. Immunofluorescence staining for αSMA and collagen was performed using anti-human αSMA antibodies (Clone 1A4, Dako, Carpinteria, CA, USA) and a fluorescent probe (CNA35) for live monitoring of collagen, as previously reported by us (35). Sections were counterstained with DAPI (Life Technologies). At 15 days the hydrogel sections were stained for αSMA, vimentin (Ab20346, Abcam), collagen type I (c2456, Sigma Aldrich) and collagen type III (Ab7778, Abcam). ALP activity was visualized with a 5-bromo-4-chloro-3-indolyl phosphate/nitroblue tetrazolium (BCIP/NBT) solution (Amresco, Kaysville, UT, USA) and an eosin counterstaining (Sigma Aldrich).

### Quantification of Alkaline Phosphatase Activity in the 2D Models

Alkaline phosphatase (ALP) activity in the 2D irradiated VICs were quantified using a colorimetric assay kit (Biovision, Milpitas, CA) on day 1, 7 and 14, and normalized to DNA content.

### Real-Time Polymerase Chain Reaction

To quantify mRNA expression of irradiated VICs, RT-PCR was performed RNA was isolated with a RNeasy kit (Qiagen, Valencia, CA, USA) on day 1, 7, and 14 after irradiation. From the extracted RNA, complementary DNA was made with oligo-(dT)12-18 primers and SuperScript II reverse transcriptase (Life Technologies). RT-PCR was performed using SYBR Green (BioRad, Hercules, CA, USA) for *ACTA2* (cell activation), *COL1A1* and *COL3A1* (cell functionality), *VIM* (mesenchymal differentiation) and the housekeeping gene glyceraldehyde 3-phosphate dehydrogenase (GAPDH). The following primer sequences were used: *ACTA2*: F:5′-AGTGCGACATTGACATCAGG-3′ and R:5′-CTGGAAGGTGGACAGAGAGG-3′; *COL1A1*: F:5′CCAAGAGGAGGGCCAAGAAGAAGG-3′ and R:5′-GGGGCAGACGGGGCAGCACTC-3′, *COL3A1*: F:5′CCTGGACGAGATGGAAACCC-3′ and R:5′-ATTTTCACCACGATCGCCCT-3′, *VIM:* F:5′-AGCAGTATGAGAGTGTGGCC-3′ and R:5′CTTCCATTTCCCGCATCTGG-3′, and *GAPDH*: F:5′-CCCAGAAGACTGTGGATGG-3′, R:5′- ACCTGGTCCTCAGTGTAGCC-3′. mRNA expression was quantified using the comparative Ct method.

### Quantification of Matrix Metalloproteinase Activity

To assess the relative difference in MMP activity, gelatin zymography was used. The hydrogels were disrupted with a mikro-dismembrator and loaded into a gelatin co-polymerized SDS-PAGE gel. SDS was removed from the gel by 2.5% Triton X-100 incubation. Subsequently, the gel was incubated in digestion buffer (50 mM TRIS, 4.8 mM CaCl_2_, pH = 8.5) overnight at 37°C for enzymatic digestion. Next the zymogram was stained for 2 hours with 0.1% (w/v) Brilliant Blue R in 4% (v/v) methanol and 10% (v/v) acetic acid in water followed by de-staining in 4% (v/v) methanol and 10% (v/v) acetic acid in water for 1 h. The zymogram was imaged with a Proxima AQ-4 scanner (Isogen Life Science). Band intensities were analyzed using ImageJ (U.S. National Institute of Health). The MMP activity was normalized to DNA content.

### Statistical Analysis

Data are presented as mean ± standard deviation (SD) unless indicated otherwise. To evaluate differences between groups, a one-way ANOVA was performed. For *post-hoc* testing, the Bonferroni test was used. *P* < 0.05 was considered as statistically significant.

## Results

### Radiation Affects Cell Viability and Proliferation in a Dose-Dependent Manner

**Figure 1:**
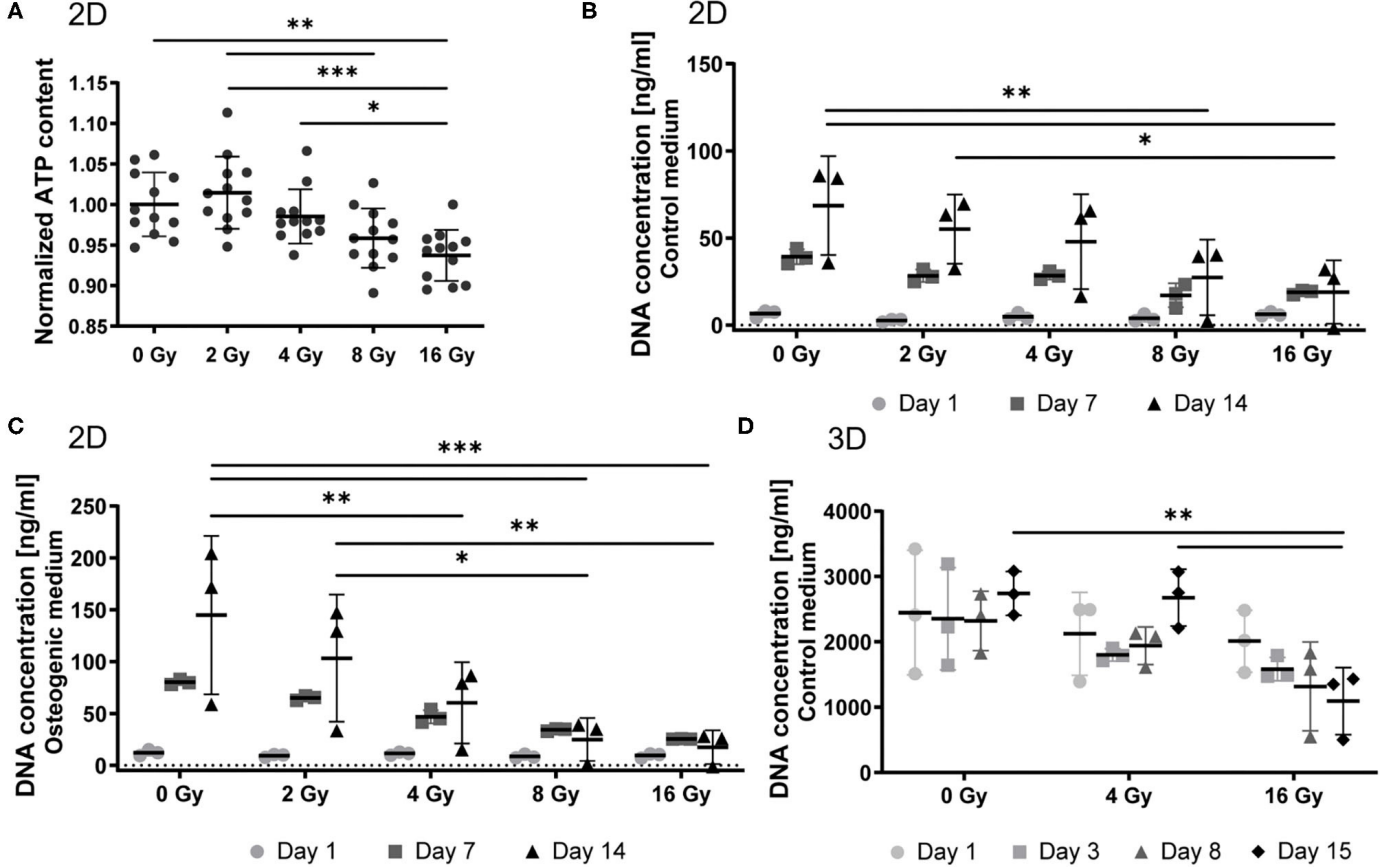
Radiation affects cell viability and proliferation in a dose-dependent manner. **(A)** Quantification of ATP content in 2D VICs exposed to 0, 2, 4, 8, and 16 Gy after 48 h and normalized for DNA content, *n* = 12. **(B–D)** Quantification of proliferation by measurement of the DNA content after 1, 7, and 14 days post radiation for 2D groups in control **(B)** and osteogenic **(C)** medium, and 1, 3, 8, and 15 days post radiation for 3D groups in control medium **(D)**. Significance is represented as *(*P* < 0.05), **(*P* < 0.01), and ***(*P* < 0.001), *n* = 3.

VICs were cultured on 2D coverslips and exposed to 0, 2, 4, 8, and 16 Gy of radiation. To evaluate cellular viability, we performed an ATP activity assay. ATP content of VICs decreased upon exposure to increasing radiation doses (Figure 1A). We observed a 7% decrease in cell viability in the 16 Gy group, compared to the control group (0 Gy). Next, we determined the proliferation response of the 2D irradiated VICs by quantifying DNA content at several time points after radiation (Figures 1B,C). The proliferative activity of these VICs decreased upon increased radiation doses, following a similar trend to cellular viability, establishing a dose-dependent response of VICs to radiation. A similar dose-dependent trend was seen in the irradiated VICs encapsulated in 3D hydrogels, whereby DNA content decreased for the 16 Gy groups 15 days post-exposure, compared to the groups that were exposed to 0 or 4 Gy (Figure 1D).

**Figure 2:**
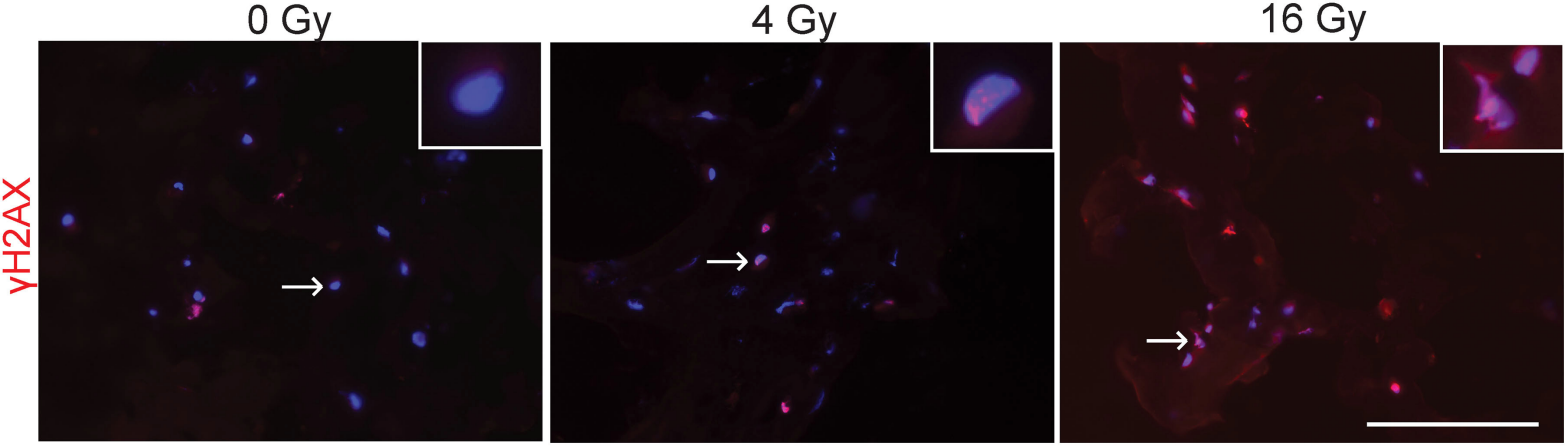
Radiation causes DNA damage. Representative immunofluorescent stained sections of 3D hydrogel samples treated with 0 Gy, 4 Gy, and 16 Gy of radiation. Samples were fixated 30–60 minutes after radiation and stained for γH2AX (red) and cell nuclei (blue). Inserts show higher magnification of cell nuclei indicated by the arrow. Scale bars represent 100 μm.

To evaluate potential DNA damaging effects of radiation exposure, 3D hydrogel sections were stained for γH2AX to detect radiation-induced double stranded DNA breaks (Figure 2). These stainings showed that in the groups exposed to 4 and 16 Gy, more γH2AX foci are present and have an increased intensity compared to the control group (0 Gy). Our results suggest that radiation causes dose-dependent DNA damage in VICs.

### Radiation Causes Myofibroblast-Like Morphological Changes and Differentiation of VICs

**Figure 3:**
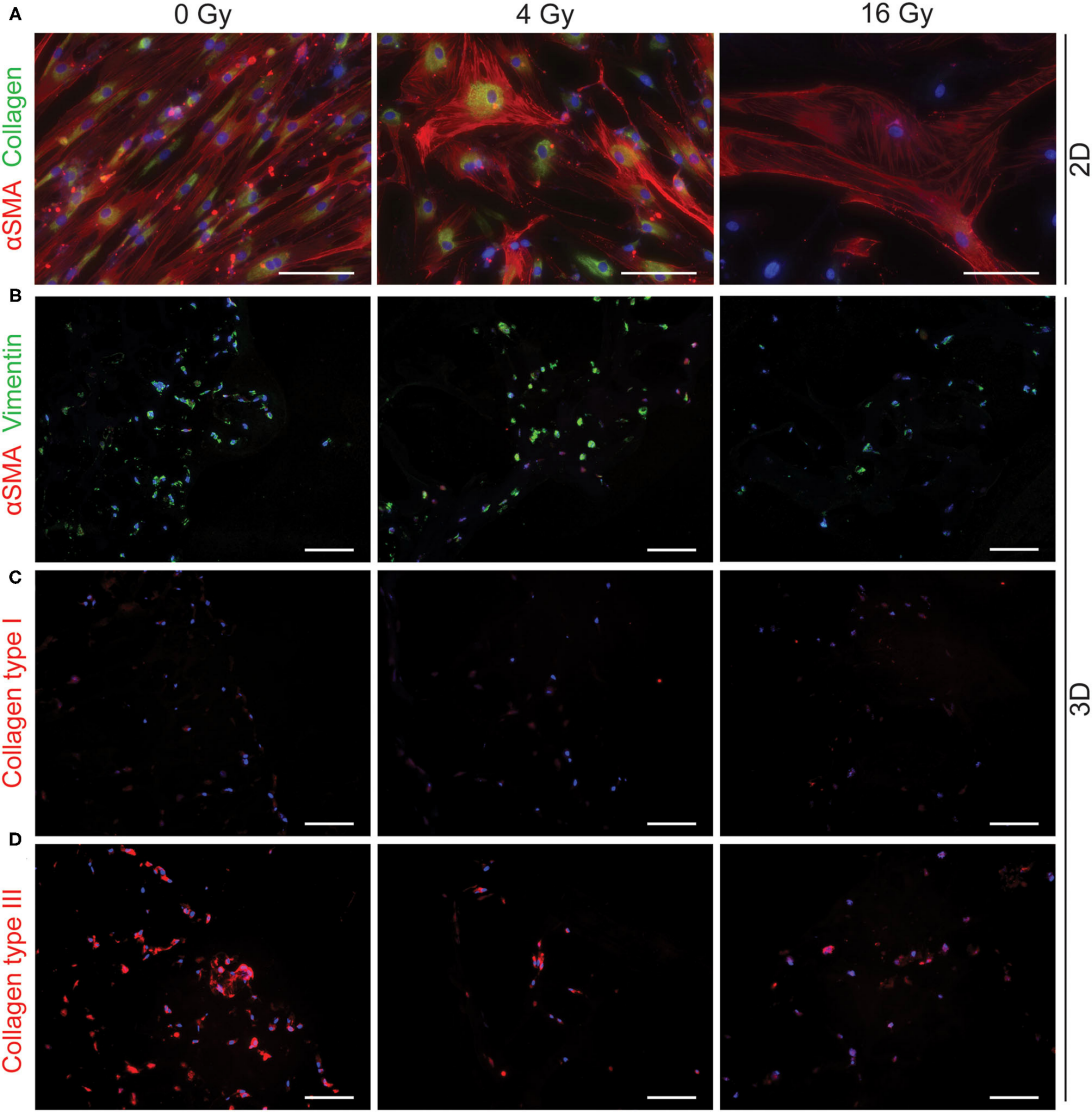
Radiation adversely affects cell morphology in control medium. Staining of 2D cell cultures 14 days after 0 Gy, 4 Gy, and 16 Gy of radiation treatment for αSMA (red) and collagen (green) **(A)**. Sections of 3D hydrogels are stained for αSMA (red) and vimentin (green) **(B)**, collagen type I **(C)**, and collagen type III **(D)**. Nuclei are visualized in blue. Scale bars represents 50 μm in 2D cultures and 100 μm in 3D cultures.

**Figure 4:**
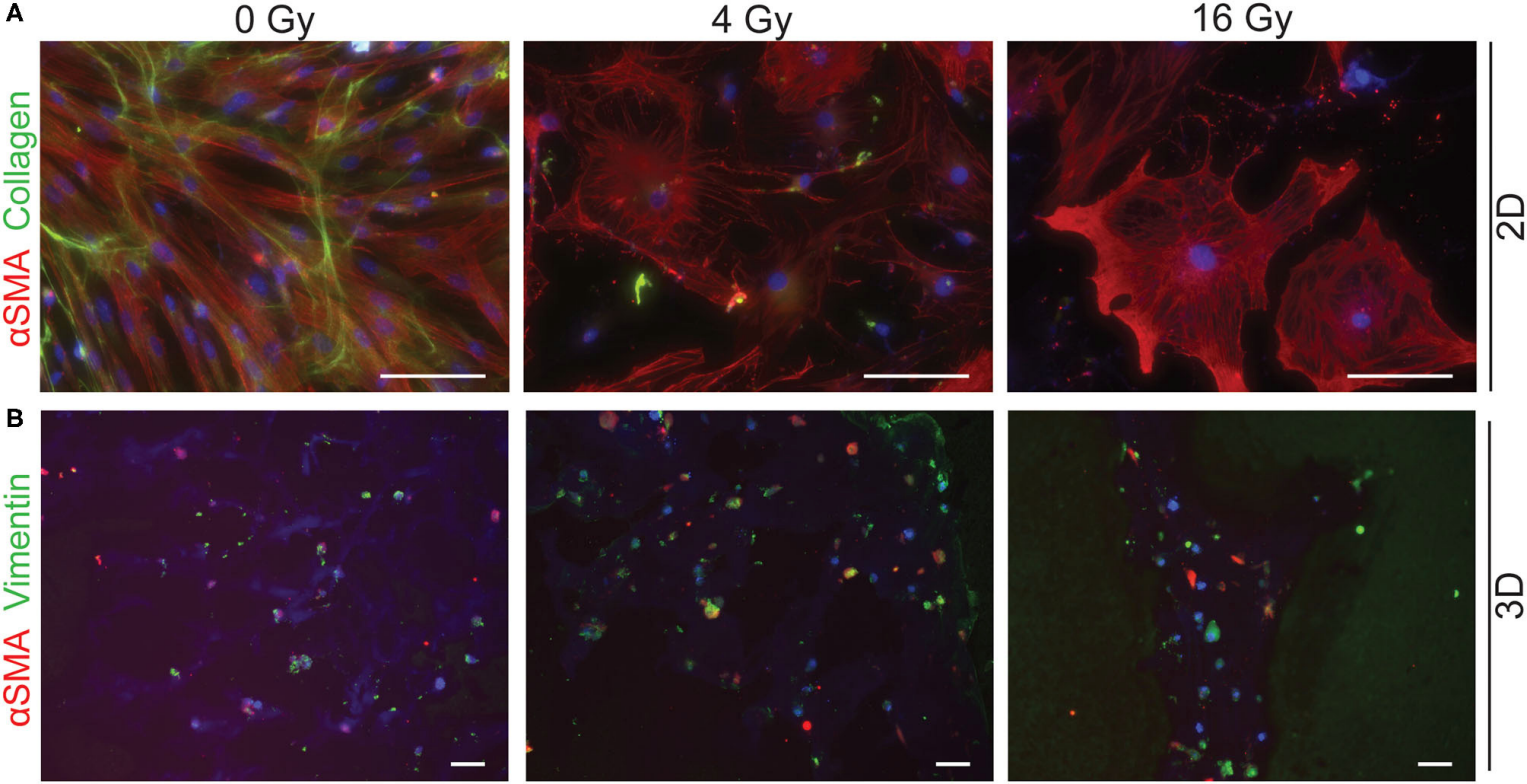
Radiation adversely affects cell morphology in osteogenic medium. Staining of 2D cell cultures for αSMA (red) and collagen (green) 14 days after 0 Gy, 4 Gy, and 16 Gy of radiation treatment **(A)**. Sections of 3D hydrogels are stained for αSMA (red) and vimentin (green) **(B)**. Nuclei are visualized in blue. Scale bars represents 50 μm in 2D cultures and 100 μm in 3D cultures.

To assess different characteristics of the 2D and 3D irradiated VICs, immunohistochemistry and gene expression analyses (RT-PCR) were performed. VICs demonstrated a myofibroblast-like phenotype when cultured in both CM (Figure 3A) and OM (Figure 4A), characterized by αSMA expression. In both conditions, the number of cells in each well decreased when exposed to increasing radiation doses. Conversely, the cellular size of VICs visually increased upon exposure to higher radiation doses. After exposure to higher radiation doses (16 Gy), VICs were visually observed to adapt a giant cell-like fibroblast phenotype (Figures 3A, 4A).

**Figure 5:**
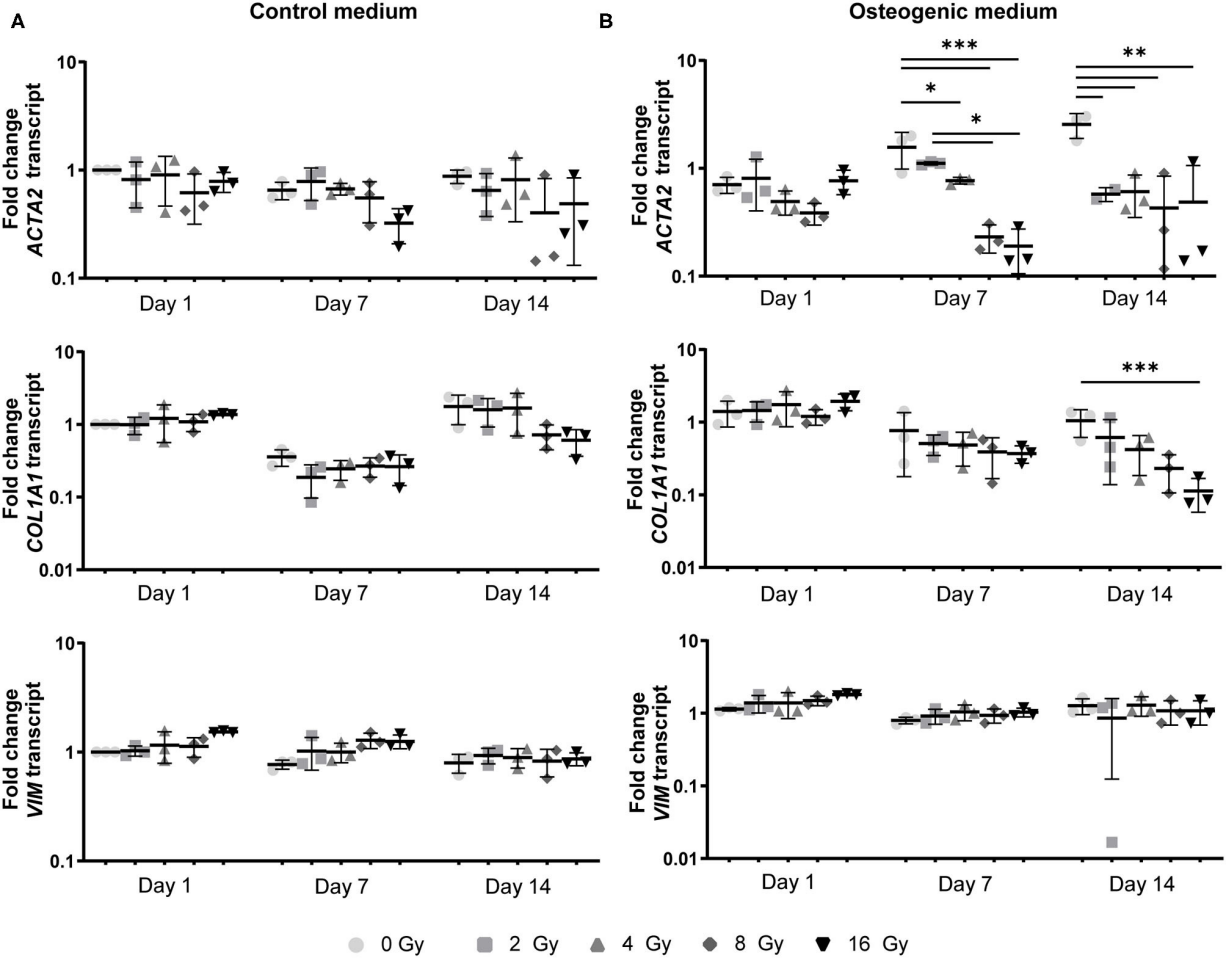
Myofibroblast-like differentiation is affected by radiation. Gene expression analysis of *ACTA2, COL1A1*, and *VIM* in VICs at 1, 7, and 14 days after radiation exposure in control **(A)** and osteogenic medium **(B)** in a 2D environment. Significance is represented as *(*P* < 0.05), **(*P* < 0.01), and ***(*P* < 0.001), *n* = 3.

**Figure 6:**
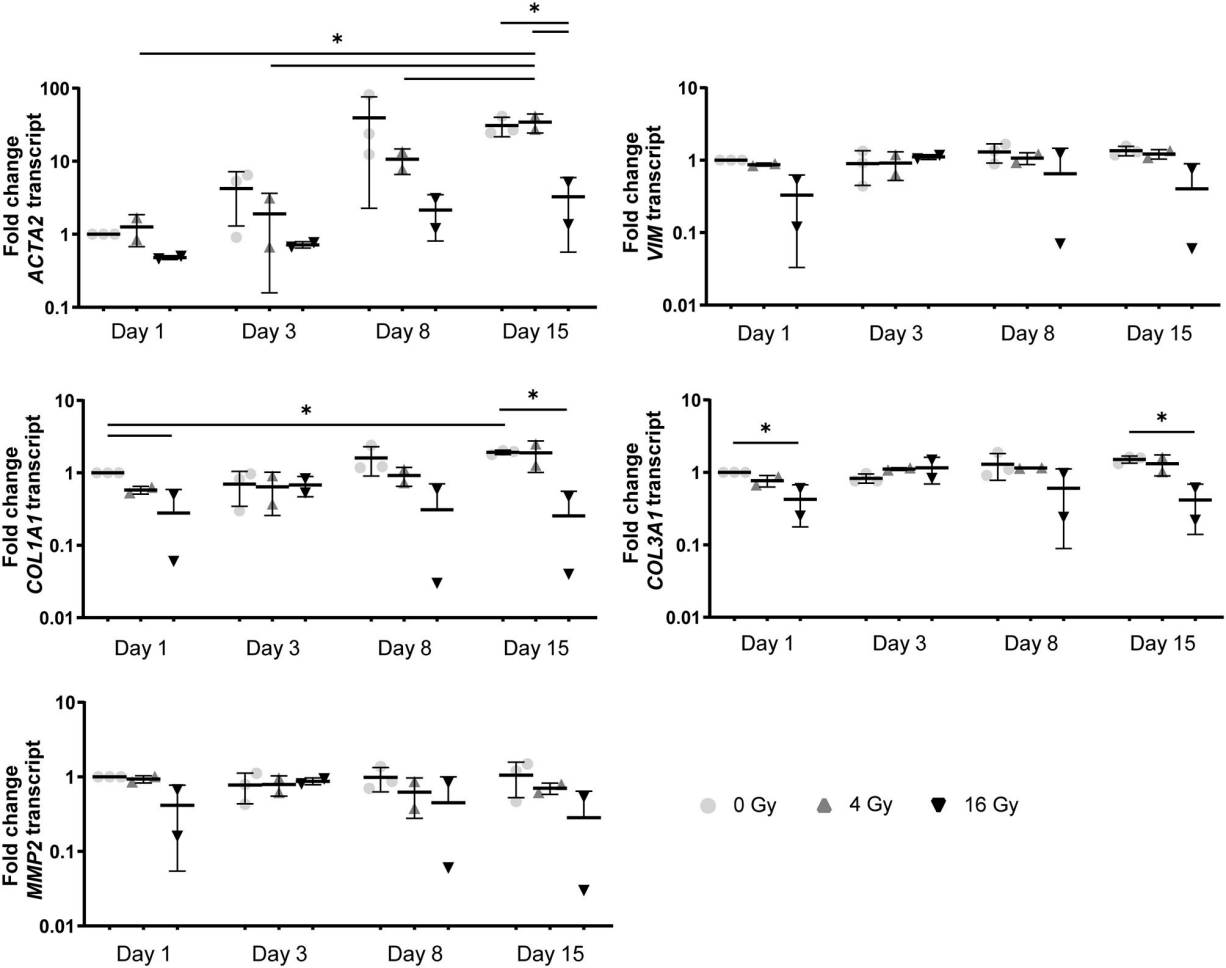
In a 3D environment radiation exposure to 16 Gy, but not 4 Gy, induces changes in VIC differentiation. Gene expression analysis *ACTA2, VIM, COL1A1, COL3A1*, and *MMP2* in VICs at 1, 3, 8, and 15 days post radiation in control medium in 3D hydrogels. Significance is represented as *(*P* < 0.05), **(*P* < 0.01), and ***(*P* < 0.001), *n* = 3.

In addition, a decrease in the number of αSMA-positive cells was observed in both CM and OM when exposed to increasing doses of radiation, compared to the control group (0 Gy) (Figures 3A, 4A). These observations were confirmed by RT-PCR, where αSMA expression followed a similar trend (Figure 5A). No differences between groups were observed at day 1 after irradiation, but a clear decrease in αSMA expression was seen at day 7 post-exposure. In the 3D hydrogels, less αSMA-positive cells were present in both culture conditions compared to the 2D VICs (Figures 3B, 4B). However, no clear difference could be observed between the three radiation doses after 15 days. Nonetheless, gene expression analysis showed that 16 Gy, but not 4 Gy, causes a significant decrease in αSMA expression in 3D irradiated VICs (Figure 6).

### Radiation Causes Osteogenesis in VICs

**Figure 7:**
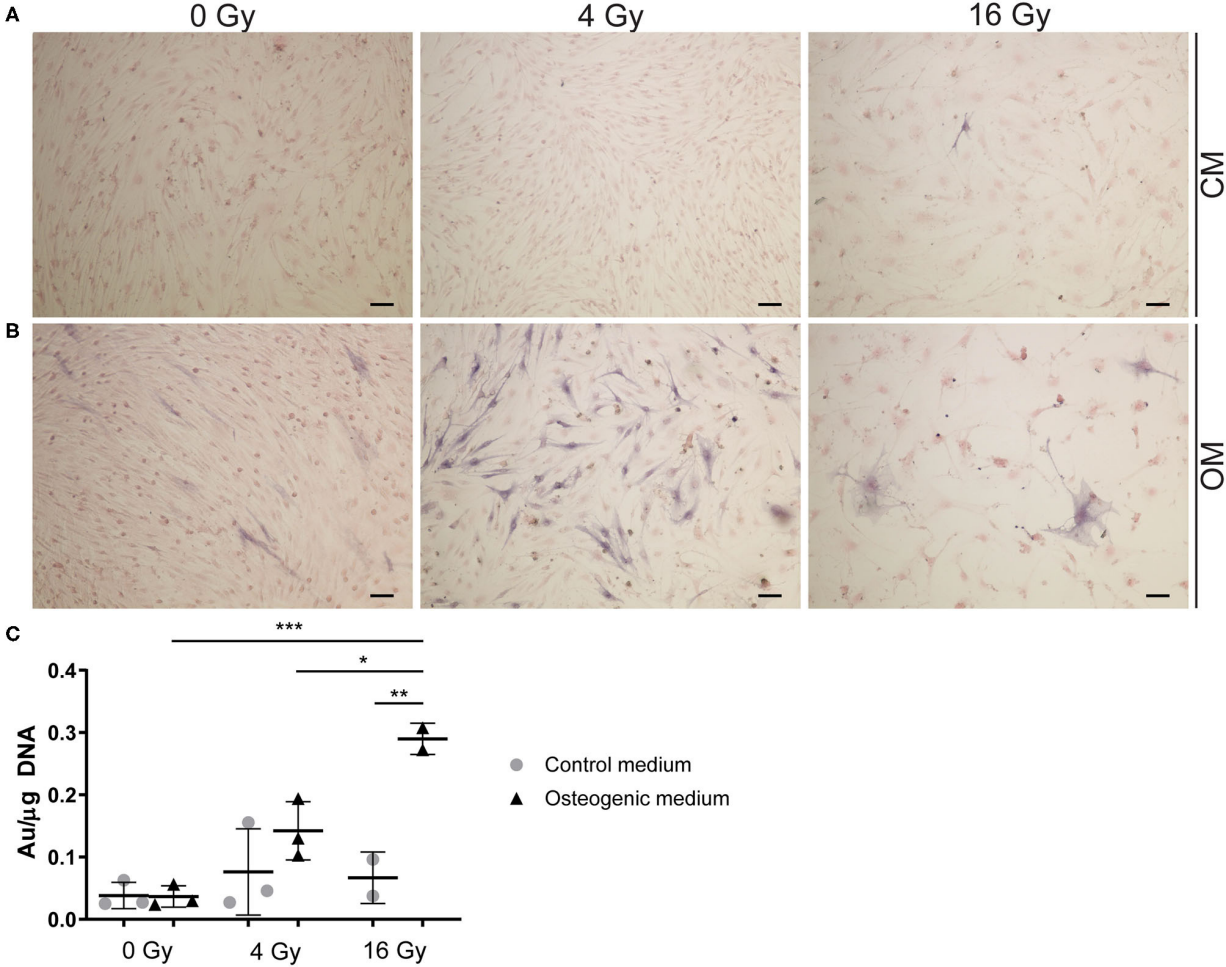
Radiation increases alkaline phosphatase activity. **(A,B)** Staining of VICs in 2D culture for ALP in dark blue and the cytoplasm in pink. Cells were cultured in control **(A)** or osteogenic **(B)** medium. For radiation treatment 0, 4, and 16 Gy were used. **(C)** Quantification of ALP activity in VICs exposed to radiation in control medium (CM) and osteogenic medium (OM). Significance is represented with *(*P* < 0.05), **(*P* < 0.01), and ***(*P* < 0.001), *n* = 3.

To assess the effects of radiation on osteogenesis in VICs, the activity of early osteogenic marker alkaline phosphatase (ALP) was measured at day 14 of cell culture. To visualize ALP activity, VICs were stained with BCIP/NBT, resulting in a dark blue staining due to ALP hydrolyzing BCIP (Figures 7A,B). We demonstrated that ALP activity is present in 2D cultured VICs at day 14 of culture and quantified ALP at that day. No ALP activity was observed in the CM groups, apart from the groups that were exposed to high radiation doses (16 Gy). However, when cultured in OM, VICs showed ALP activity predominantly in the group exposed to 4 Gy. Upon exposure to 16 Gy, less ALP-positive cells were observed (Figure 7C).

### Matrix Turnover in 3D Hydrogels Changes After Radiation Exposure

**Figure 8:**
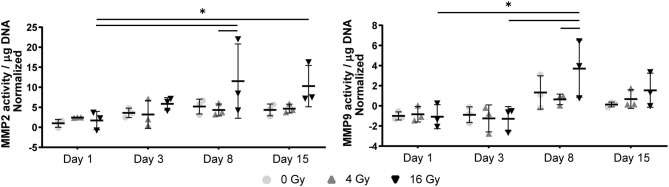
MMP activity in 3D hydrogels increases with higher radiation dose and with time. Quantification of matrix metalloproteinase (MMP) 2 and 9 activity in the hydrogels at day 1, 3, 8, and 15 post radiation. MMP activity is normalized to background activity from the medium in cell-free hydrogels. Significance is represented with *(*P* < 0.05), **(*P* < 0.01), and ***(*P* < 0.001), *n* = 3.

In the 2D irradiated VICs, we observed a decrease in collagen staining at day 14 of culture, which remained present in a lesser amount after irradiation with 4 Gy but was obsolete at higher radiation doses (Figures 3A,C,D). This was confirmed by gene expression of collagen type 1 (*COL1A1*), which showed a decline in expression alongside increasing radiation doses, both in 2D culture (Figure 5B) and in the 3D hydrogels (Figure 6). In the 3D hydrogels, MMP2 and MMP9 activity also increased over time with higher radiation doses (Figure 8).

## Discussion

The presented work focused on studying mechanisms of radiation-associated valvular disease using both 2D and 3D established *in vitro* platforms to study CAVD (33, 36). We demonstrated that *in vitro*, radiation exposure induces a decrease in cell viability and proliferation of cultured VICs in a dose-dependent manner. In addition, we showed decreased myofibroblast-like differentiation of VICs when exposed to increasing radiation doses, both in control and osteogenic media. However, an increase in early osteogenic activity was observed upon increasing radiation doses in an osteogenic environment. Next, we visually observed morphological transformation of VICs into a giant cell-like fibroblast phenotype after exposure to higher doses of radiation in osteogenic medium. Finally, we showed that ECM remodeling was affected by radiation exposure. While deposition of new ECM proteins, such as collagen type I and III, was decreased after radiation exposure, MMP activity was induced. In sum, these results suggest that radiation exposure catalyzes the calcific response of VICs as observed in CAVD.

However, these outcomes should be interpreted carefully. After exposure of 2D and 3D VICs to higher doses of radiation (8 and 16 Gy), we observed not only a decreased viability and proliferation, but also a marked change in cell morphology as was apparent by the giant fibroblast-like aspect they obtain after 14 days of culture. Previous studies have reported on these atypical giant-sized fibroblasts in skin, indicating their terminal differentiation (28). Although we have not evaluated DNA damage at this stage, other work has demonstrated that high doses of radiation are associated with increased cellular damage (37) that could suggest the abnormal morphological changes of the VICs in our work. Importantly, these abnormal cells exhibited ALP activity, a marker for early osteogenesis (38). Additional analysis of the mineralization potential of these giant cells is still warranted, but our results already indicate that radiation may induce an osteogenic VIC phenotype *in vitro*. VICs have a higher radio-resistant capacity than most cancer cell lines (39). Nevertheless, they do follow a similar dose-dependent pattern to other cancer cell line studies (39). Osteogenic activity was only observed in VICs cultured in osteogenic medium and exposed to high doses of radiation, supporting our hypothesis that radiation accelerates the osteoblast-like differentiation of VICs, but only when exposed to an osteogenic environment.

VICs are essential in maintaining structural and functional integrity of the aortic valve by continuous remodeling of the ECM through deposition of matrix components, such as collagen, and matrix degradation through increased MMP activity. Our results indicate that ECM remodeling is significantly affected by radiation exposure. The data show that the deposition of newly formed collagen was decreased with exposure to higher radiation doses. On the other hand, MMP-2 and MMP-9 proteolytic activity was increased with increasing radiation doses, suggesting that matrix degradation does continue to occur. The elevation of MMP activity after radiation exposure has already been extensively reported in patients receiving radiotherapy for various types of cancer, including breast cancer and malignant gliomas (40–42). Our findings are therefore in line with other studies that examined the effects of radiation on MMP function. However, further studies are needed to unravel the role of these proteases on the pathogenesis and progression of radiation-associated valvular disease. These findings also emphasize the importance of using 3D models in this context, since these models enable the possibility to study the interplay between VICs and their environment.

Few reports have investigated radiation-associated CAVD (27, 32). Studying the VIC response in conventional *in vitro* models is not sufficient to recapitulate all events that may occur *in vivo*. To overcome this challenge, hydrogel micro-engineering has emerged as a powerful tool to create 3D tissue models recapitulating the VIC micro-environment *in vitro*. We have previously demonstrated that combining hyaluronic acid and collagen by UV photo-crosslinking, a 3D environment can be created that mimics the micro-environment of the aortic valve (33, 36). Since this system facilitates VIC quiescence, it could offer a valuable platform to study the effects of radiation on valvular tissue as it may occur *in vivo*, since culturing VICs on stiff culture plates has been associated with myofibroblastic activation of the cells (33). Our results demonstrate that the hydrogel structure seems to be unaffected by radiation exposure, as measured by changes in compaction. Possibly, elasticity and porosity of the hydrogels should also be evaluated in future assays. Moreover, the VICs that were being irradiated in this study were quiescent before irradiation, which more accurately mimics *in vivo* conditions than when VICs are cultured on stiff culture plates and thus already become activated prior to irradiation.

Because of our observations that VICs in culture develop into atypical giant cell-like fibroblasts when exposed to higher doses of radiation (8 and 16 Gy), but retain their native morphology at 4 Gy, we chose to expose the VIC-laden hydrogels to only lower and higher doses (4 and 16 Gy, respectively). Although radiotherapy is mostly conducted in a fractionated manner, 4 Gy is within the therapeutic range that patients undergoing radiotherapy receive. In addition, exposure to 16 Gy caused VICs to express osteogenic activity even in normal growth medium, potentially accentuating the accelerated mineralization observed in patients.

In non-radiation associated CAVD, mechanical stress is thought to be one of the initiators of calcification of the valve (43). This type of stress can induce lesions in the fibrosa layer of the aortic valve, eventually leading to the development of CAVD (18). Radiation can also cause microfractures in collagen (44). Therefore, such microlesions in irradiated heart valves can be one of the key mechanisms in valvular heart disease after radiation therapy. Although compaction analysis showed no effect of radiation on the hydrogels, it is not ruled out that there is damage to the ECM proteins in the hydrogel. Structural analysis of the hydrogels exposed to radiation is therefore needed to investigate these possible mechanisms.

To date, little is known about the relationship between VICs and radiotherapy. To our knowledge, only one other study examined the effects of radiation exposure on VICs. Nadlonek et al. demonstrated an increase in osteoblast-like differentiation of VICs *in vitro*, when exposed to radiation (32). However, only one dose of radiation was used in this study. In order to further adjust dose regimens and protocols of radiotherapy in the future, it is essential to consider the variation of effects radiotherapy may have on tissue. Over the past few decades, great progress has been made in the context of radiotherapy effectiveness and safety (3, 4, 30, 31). With current standards, gamma radiation can better focus on targeted tissue and surrounding tissues can be spared from the damaging effects of radiation. Because of these developments, it is now possible to use higher doses of radiation targeted to a smaller area. However, these new techniques such as inverse-planned intensity modulated radiation therapy (IMRT), used for instance in breast cancer treatment, still involve radiation beams passing through the heart and exposing it to radiation (45, 46). In addition, indirect damage by reactive oxygen species (ROS) as a consequence of irradiation may occur in otherwise shielded areas (47, 48). Radiation-induced valvular disease remains an important issue in patients receiving radiotherapy.

We hypothesized that radiation accelerates the calcific response in the aortic valve. This work demonstrates that radiation exposure enhances loss of the myofibroblast-like phenotype of VICs and promotes their mineralization activity. In addition, we demonstrate that ECM remodeling, one of the most important functions of VICs in maintaining homeostasis of the aortic heart valve, is severely affected by radiation exposure. This might explain the late onset valvular deterioration that is seen in patients decades after receiving mediastinal radiotherapy. Lastly, this work emphasizes the importance of tissue-engineered 3D models of the valvular microenvironment for understanding key processes of radiation-associated CAVD, as they may occur *in vivo*. Further research using such models in direct comparison with *in vivo* data is required to draw clinically relevant conclusions on the development and progression of radiation-associated CAVD of the human aortic valve.

## Data Availability Statement

The original contributions presented in the study are included in the article/Supplementary Material, further inquiries can be directed to the corresponding author/s.

## Ethics Statement

Ethical review and approval was not required for the animal study because in this work, porcine valvular interstitial cells (VICs) were used from rest material from pigs sacrificed at a slaughterhouse. Therefore, it was not necessary to obtain ethical approval from an ethics committee upon using these cells.

## Author Contributions

JH, RD, NE, and CB designed the experiments and RD, NE, JH, and IB performed the experiments. JS performed the radiation in the Catharina Hospital, Eindhoven. Data analysis, interpretation and creation of figures was performed by MM, RD, NE, IB, JH, EA, CB, and DK. The manuscript was written by MM, RD, NE, and JH. JH and CB supervised the project and secured funding. All authors have read, commented, and agreed to the content of the manuscript.

## Conflict of Interest

The authors declare that the research was conducted in the absence of any commercial or financial relationships that could be construed as a potential conflict of interest.

## Publisher's Note

All claims expressed in this article are solely those of the authors and do not necessarily represent those of their affiliated organizations, or those of the publisher, the editors and the reviewers. Any product that may be evaluated in this article, or claim that may be made by its manufacturer, is not guaranteed or endorsed by the publisher.

## Supplementary Material

Supplemental Figure 1 Gelatin zymography bands for active MMP-9 (82 kDa) and active MMP-2 (62 kDa) measured 1, 3, 8 and 15 days post radiation (with 0 Gy, 4 Gy or 16 Gy). Three donors were included for every condition and non-radiated empty hydrogels (without VICs) were used as a negative control.

## Abbreviations

ALP: alkaline phosphatase
αSMA: alpha smooth muscle actin
ATP: adenosine triphosphate
CAVD: calcific aortic valve disease
CM: control medium
ECM: extracellular matrix
GelMA: methacrylated gelatin
Gy: Gray
HAMA: methacrylated hyaluronic acid
MMP: matrix metalloproteinase
OM: osteogenic medium
RT-PCR: real-time polymerase chain reaction
VIC: valve interstitial cells
Runx2: runt-related transcription factor 2
TGFβ: transforming growth factor beta.
