# Supplementary figures and images for: Radiation Induces Valvular Interstitial Cell Calcific Response in an in vitro Model of Calcific Aortic Valve Disease
Source: Front Cardiovasc Med. 2021 Aug 30;8:687885. doi: 10.3389/fcvm.2021.687885 (PMC8435633; doi:10.3389/fcvm.2021.687885)

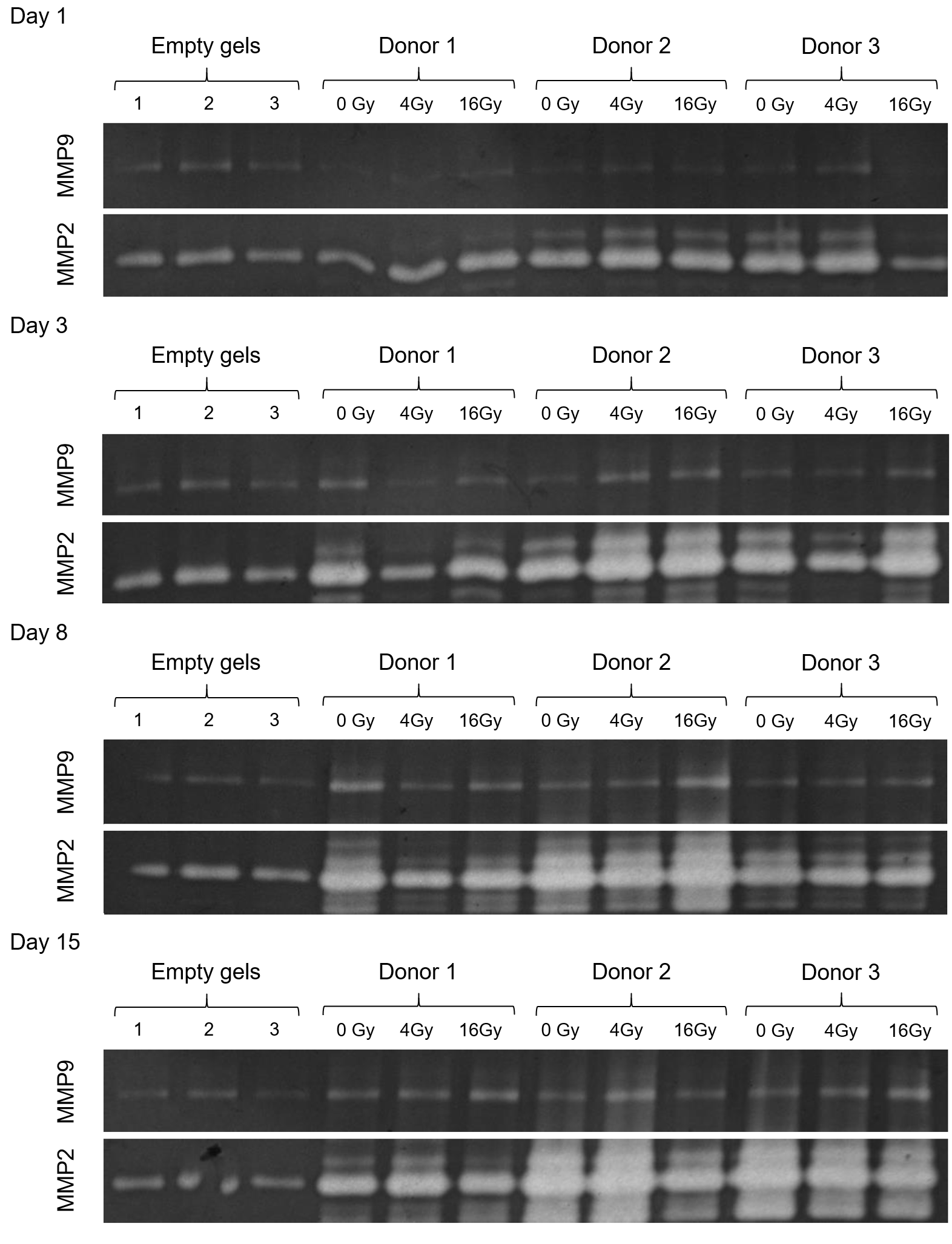

Supplement: Supplemental Figure 1 — Gelatin zymography bands for active MMP-9 (82 kDa) and active MMP-2 (62 kDa) measured 1, 3, 8 and 15 days post radiation (with 0 Gy, 4 Gy or 16 Gy). Three donors were included for every condition and non-radiated empty hydrogels (without VICs) were used as a negative control. [file Image_1.tif]
